# The Two-Faced Role of crAssphage Subfamilies in Obesity and Metabolic Syndrome: Between Good and Evil

**DOI:** 10.3390/genes14010139

**Published:** 2023-01-04

**Authors:** Melany Cervantes-Echeverría, Luigui Gallardo-Becerra, Fernanda Cornejo-Granados, Adrian Ochoa-Leyva

**Affiliations:** Departamento de Microbiologia Molecular, Instituto de Biotecnologia, Universidad Nacional Autonoma de Mexico, Avenida Universidad 2001, Cuernavaca 62210, Morelos, Mexico

**Keywords:** crAssphage, crAss-like virus, obesity, metabolic syndrome

## Abstract

Viral metagenomic studies of the human gut microbiota have unraveled the differences in phage populations between health and disease, stimulating interest in phages’ role on bacterial ecosystem regulation. CrAssphage is a common and abundant family in the gut virome across human populations. Therefore, we explored its role in obesity (O) and obesity with metabolic syndrome (OMS) in a children’s cohort. We found a significantly decreased prevalence, diversity, and richness of the crAssphage Alpha subfamily in OMS mainly driven by a decrease in the Alpha_1 and Alpha_4 genera. On the contrary, there was a significant increase in the Beta subfamily in OMS, mainly driven by an increase in Beta_6. Additionally, an overabundance of the Delta_8 genus was observed in OMS. Notably, a decreased abundance of crAssphages was significantly correlated with the overabundance of Bacilli in the same group. The Bacilli class is a robust taxonomical biomarker of O and was also significantly abundant in our OMS cohort. Our results suggest that a loss of stability in the Alpha subfamily of crAssphages is associated with O and OMS. Contrary, an overabundance of the Delta subfamily was found in OMS. Our study advises the importance of considering the dual role (good and evil) of crAssphage subfamilies and their participation in conditions such as O, where we suggest that Alpha loss and Delta gain are associated with obese individuals.

## 1. Introduction

Recent advances in bioinformatics and experimental protocols for viral metagenomics have shown that the gut virome is mainly dominated by bacteriophages [1,2]. Indeed, there are disease-specific changes in the gut virome in inflammatory bowel disease [3,4], AIDS [5], diabetes [6], malnutrition [7], obesity, and obesity with metabolic syndrome [8]. There are many reasons to study the relationship between phages and microbiota, including the fact that they kill specific bacteria and transfer different genes; consequently, they can alter the host’s relationship with the microbiota [9]. Human feces have been estimated to contain 10^9^ VLPs per gram of feces [8]. Although, there are also “passenger viruses” that are diet-associated, such as plant and dietary viruses [10].

In 2014, Dutilh et al. identified crAssphage from the unknown viral fraction of the human gut microbiota via cross-assembly, and based on the co-occurrence of the host and CRISPR spacer profile, crAssphage was predicted to infect the bacterial members of the Bacteroidetes phylum [11]. Since their discovery, crAssphages have been one of the most intriguing viruses within the human gut virome [11,12], being one of the most abundant and common components of the gut virome across the human population [13,14]. In addition, they have considerable genomic diversity and appear to have coevolved with humans [13,15]. Further, some sequence-based analyses showed that this bacteriophage family has a combination of lytic and lysogenic lifestyles encoded into their genomes [12,16].

In 2018, Guerin et al. used polymerase and/or protein terminase sequences as a genetic signature to identify crAss-like phages and defined their taxonomic ranks in subfamilies Alphacrassvirinae, Betacrassvirinae, Gammacrassvirinae, and Deltacrassvirinae that are distributed in 10 genera [15].

Additionally, several crAssphages are associated with non-human primate populations that are globally distributed [13]. They are abundant and widespread in diverse animal and environmental human habitats [12]. Indeed, despite the high interindividual variations in the gut virome [8], the crAssphages constitute a global virus marker for global-scale studies [13], being part of the core human virome [17].

The crAssphages are generally scarce in the feces of one-month-old infants, increasing their abundance at four months old and in 2–5-year-old toddlers, and becoming high-level persistent in adults [18]. Indeed, these phages are detected in both the mother and the infant, suggesting vertical transmission, and they also can be acquired through fecal microbiota transplantation [19]. The consistently high prevalence of crAssphage across most human samples has led to the proposal of this bacteriophage as an indicator of human fecal pollution, for example, for monitoring water quality [20,21].

Moreover, in 2014, Dutilh et al., using sequence analyses, published the first study that suggested *Bacteroides* as a potential host [11]. Later, in 2018, Shkoporov et al. reported the isolation of a crAssphage from human fecal samples and corroborated that it infected *Bacteroides intestinalis*, confirming previous in silico predictions of the likely host [22]. Finally, current research has proposed other potential hosts, such as Prevotella, Collinsella, Bacilli, and Eubacterium [11,19].

Recent studies have focused on estimating the association of crAssphage with lifestyle factors and different diseases. For example, a study that analyzed more than 3000 samples of human gut metagenomic data observed a low prevalence of crAssphage in traditional hunter–gatherer populations compared to industrialized urban populations. However, there were no associations with the health, age, sex, or body-size variables [13,23]. Additionally, some studies suggested that CrAssphage abundance showed no association with diseases such as diarrhea, Crohn’s disease (CD), human immunodeficiency virus (HIV), inflammatory bowel disease (IBD), type-2 diabetes (T2D), and malnutrition [15,16,23]. On the contrary, two studies that sequenced total DNA and analyzed the assembled contigs showed its associations with ulcerative colitis (UC) and colorectal cancer.

The first study showed that the abundance of crAssphages had decreased in individuals with colorectal cancer without alteration of putative host genera *Bacteroides*, suggesting that therapy through fecal CrAssphage transplantation could be a potential treatment to restore their abundance [24]. Moreover, the second study described that members of the crAssphage family were significantly higher (23.5%) in patients with ulcerative colitis (UC) compared to non-diseased patients (2.51%). Interestingly, in this study, the authors found a decreased abundance of the putative host genus *Bacteroides* when compared to healthy controls [25]. The study of this expansive group of viruses is still in its infancy, and the fundamental features of these major players in the human virome and their actual spread in the biosphere remain to be studied [17].

Here, we explored the role that crAssphages have in obesity (O) and metabolic syndrome (OMS). Further, we evaluated crAssphage abundance as a function of gut bacteria and the anthropometric and biochemical parameters that are typically altered in O and OMS.

## 2. Materials and Methods

### 2.1. Acquisition of Viral Data and Mapping to the crAssphage Genome Database

The metagenomic data of the gut VLPs samples were obtained from a previous report by our group [8]. Briefly, we analyzed the stools of 10 normal-weight (NW), 10 obese (O), and 8 obese with metabolic syndrome (OMS) children aged 7–10 years old. All children came from households with a middle economic class income and belonged to a similar sociocultural status. They all lived in Mexico City at the time of collection and did not practice any sport regularly.

From the quality-filtered data set, 1000 random subsamplings of 700,000 paired reads were carried out per sample and used for further analysis to maintain the same sequence depth for all samples. Six samples were eliminated because they needed to meet this minimal number of reads. Thus, each set of reads from each of the 1000 subsampling exercises per sample was mapped (using SMALT) to the reference crAssphage genome database consisting of 280 non-redundant crAssphage genomes, including 248 contigs previously validated by Guerin et al., 2018, 30 crAss-like contigs identified by Yutin et al., 2018, the Mexican-crAssphage genome [14], and the first crAssphage genome assembled by Dutilh et al., 2014 (Table S1). Additionally, we added five non-intestinal phage genomes as negative controls for the read mapping (Table S2). The parameters used were 70% identity and a minimum of 60 nucleotides of the query read length covered by the k-mer word seeds. All genomes were concatenated in one multifasta archive for the read mapping simultaneously. Finally, 22 samples were selected for further analysis (Table S3). In this manner, we obtained 1000 tables of read mapping per sample, which were averaged to obtain a final table of read counts per sample. This table was then imported into R for the abundance statistical analyses of the crAssphages. Thus, the relative abundance of reads mapped to each genome was generated per sample. The statistical significance was measured using the Wilcoxon test. For taxonomy analysis, we used the four subfamilies and 10 genera proposed by Guerin et al., 2018 [15].

### 2.2. crAssphages α- and β-Diversities

The α diversity metrics were determined using the normalized reads-count table for each sample. To this end, the α-diversity metrics were determined for each 1000 subsampling exercises of 700,000 paired reads each, and the average was used as the final value for each sample. To contrast the groups, a Mann–Whitney–Wilcoxon test was performed. β-Diversity analyses were performed using the normalized reads-count table, using the Jaccard and Bray–Curtis metrics implemented in QIIME (v1.9).

### 2.3. Association of crAssphage Abundance with Metadata Variables and Microbiota

We used the previously published biochemical and anthropometric data for a comparison with the crAssphage abundances [8]. The associations between the crAssphage abundances and the biochemical, anthropometric and microbiota data were performed using the Spearman coefficient test in R using cutoffs of R > 0.7 and *p*-value < 0.005. For the abundance of microbiota, we used the data of the relative frequency of the 27 significant taxa previously reported between NW, O, and OMS in the same cohort [26].

### 2.4. Correlation of crAssphage Abundance with Their Bacterial Host

From the relative frequency of the microbiota analysis published for the same cohort [8], we selected the abundance of the following taxa: Bacteroidetes, Bacteroidia, Bacteroidales, Prevotellaceae, Prevotella, Collinsella, Bacteroides, Bacilli, and Eubacterium, which were suggested as the bacterial hosts of crAssphages. After that, we correlated them with the average abundance of crAssphage in all our samples using the Spearman coefficient test in R with cutoffs of R > 0.7 and *p*-value < 0.005.

## 3. Results

### 3.1. Decreased Abundance and Prevalence of crAssphages Were Detected in the O and OMS Groups

We first analyzed the abundance and prevalence of all crAssphage genomes among the samples using the viral metagenomics sequencing data of 28 fecal samples from a previously described cohort of 7–10-year-old children: 10 with normal-weight (NW), 10 with obesity (O), and 8 with obesity and metabolic syndrome (OMS) [8] (Table S3). Because the sequencing depth strongly influences the crAssphages genomic abundances, we normalized the sequencing depth to be the same for all the samples. To this end, 1000 sets of random subsamplings of 700,000 paired reads were generated per sample (see methods). Only seven samples from the NW group, nine from group O, and six from the OMS group met the sequencing depth requirements to be subsampled (Table S3). Afterwards, each set of random subsampled reads was mapped against the crAssphages genome database composed of 280 non-redundant genomes (Table S1, see methods). In this manner, we obtained the number of mapped reads for each crAssphage genome as the average of the 1000 random subsamplings per sample. Finally, this average normalized read-counts table was used for further analysis. From the 280 crAssphages, 208 genomes contained mapped reads, and their relative abundance ranged from 0.0011 to 3.654% of the total reads per sample (Table S4).

**Figure 1:**
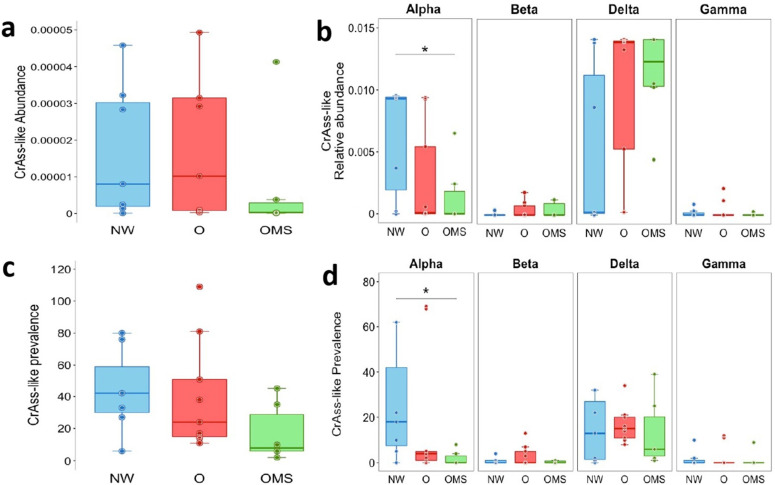
crAssphage abundance and prevalence. (**a**) The abundance of the crAssphage family. (**b**) The abundance of crAssphage subfamilies. (**c**) Prevalence of crAssphage family. (**d**) Prevalence of crAssphage subfamilies. Each point represents each sample’s average abundance or prevalence, with boxes showing the group’s distribution. * *p* < 0.05.

On the one hand, we analyzed the abundance of all the crAssphage family and found a trend for a decreased abundance in OMS compared to NW and O (Figure 1a); however, the differences were not significant. Thus, we analyzed the abundance separating each crAssphage subfamily and found a significantly decreased abundance of the Alpha subfamily (*p* = 0.047) (Figure 1b).

On the other hand, we also analyzed the prevalence of all crAssphage genomes, estimated as the number of crAssphage genomes in each sample. We found a decreased prevalence of the crAssphage family in OMS and O compared to NW (Figure 1c), although, the differences were not significant. Importantly, when comparing the prevalence of each subfamily, we found a significantly decreased prevalence for the Alpha subfamily in the OMS group compared to NW (Figure 1d).

**Figure 2:**
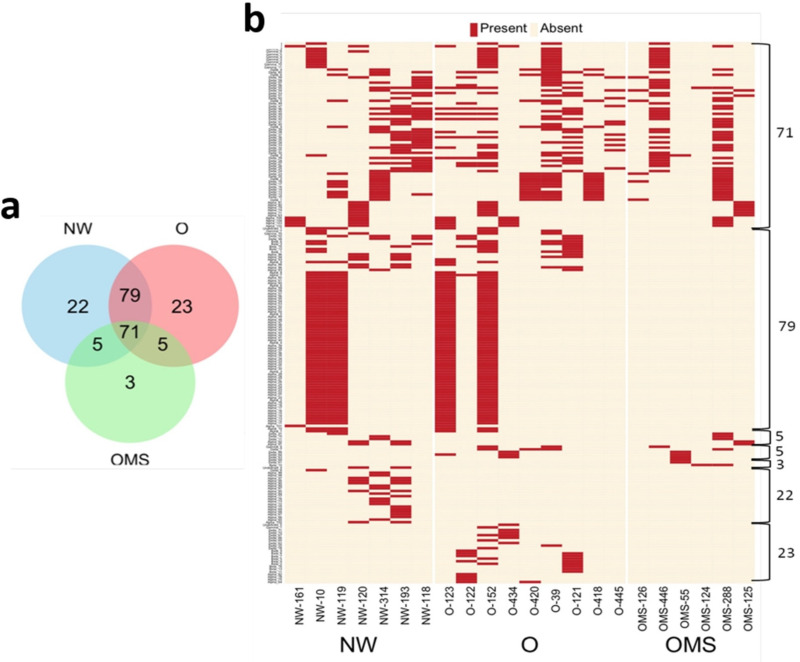
(**a**) Venn diagram of the presence of crAssphage genomes. (**b**) Presence-absence heatmap of crAssphage genomes among all samples, the number of crAssphage genomes from the Venn diagram is marked on the right.

To know the crAssphages individuality among the samples, we examined which genomes were shared among the three groups and observed the 71 phages shared among the three groups. Additionally, several phages were unique for each group (Figure 2a). The presence/absence of the genomes is presented in Figure 2b for each sample.

### 3.2. Changes in crAssphage Taxonomy Characterize the O and OMS Groups

**Figure 3:**
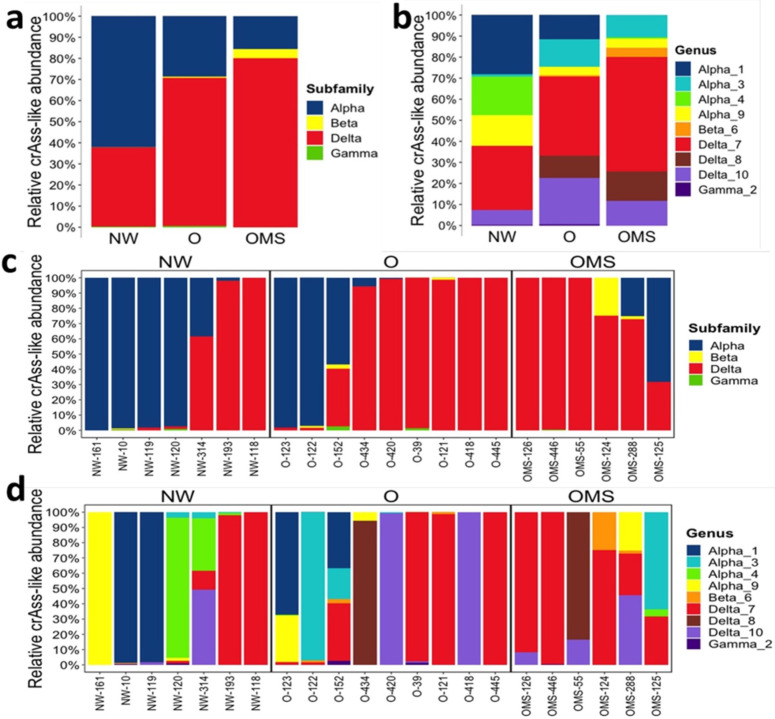
Relative abundance of crAssphage genomes grouped by taxonomy. (**a**) The average subfamilies and (**b**) genus per group; (**c**) the average subfamilies, and (**d**) genus per sample.

We examined the relative abundance of the four subfamilies and 10 genera proposed by Guerin et al., 2018 using the normalized reads-count table for each group. The Alpha and Delta subfamilies were the most abundant among the three groups (Figure 3a,c). The Alpha was significantly (*p* ≤ 0.01) enriched in NW (mean= 0.00592) when compared to O (mean = 0.00274) and OMS (mean = 0.00148). The Beta subfamily was significantly enriched in OMS (mean = 0.00298) when compared to O (mean= 0.00040) (*p* ≤ 0.05) and NW (mean = 0.00006) (*p* ≤ 0.05). An increased abundance for Delta was also observed in OMS (mean = 0.01126) and O (mean = 0.00989) when compared to NW (mean = 0.00529); however, the difference was not significant.

At the genus level, Delta_7 represented the most abundant phages among the three groups; however, no significant difference was observed (Figure 3b,d). On the contrary, Alpha_1 showed a significant (*p* ≤ 0.01) increase in NW (mean = 0.00454) when compared to O (mean = 0.00187) and OMS (mean = 0), and Alpha_4 also showed a significant (*p* ≤ 0.01) increase in NW (mean = 0.01013) when compared to O (mean = 0.00003) and OMS (mean = 0.00047). Furthermore, Beta_6 abundance showed a significant increase in OMS (mean = 0.00298) when compared to O (mean = 0.00040); however, when compared to NW (mean = 0.00006), the difference was not significant. Finally, Delta_8 showed a significant (*p* ≥ 0.01) increase in OMS (mean = 0.01263) when compared to O (mean = 0.00953) and NW (mean = 0). Interestingly, the phages from genus Gamma_5 were not present in any sample, while Delta_8 and Alpha_1 were depleted in NW and OMS, respectively.

### 3.3. Decreased Diversity and Richness of α crAssphages Are Associated with the O and OMS Groups

**Figure 4:**
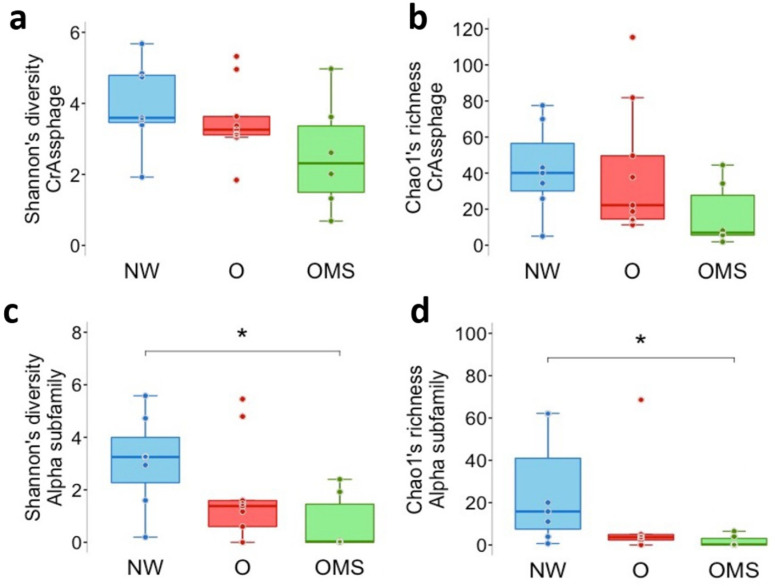
α diversity of crAssphage genomes and relative abundance of bacterial crAssphage hosts. (**a**) crAssphage diversity. Each point shows the mean of the normalized read counts for the Shannon diversity of each sample, with the boxes showing the group’s distribution. (**b**) crAssphage richness. Each point shows the mean of the normalized read counts of the observed genomes for each sample, with the boxes showing the group’s distribution. (**c**) Diversity of α subfamily. Each point shows the mean of the normalized read counts for the Shannon diversity for each sample, with the boxes showing the group’s distribution. (**d**) The richness of the Alpha subfamily. Each point shows the mean of the normalized read counts of the observed genomes for each sample, with the boxes showing the group’s distribution. Error bars represent the median ± SD. * *p* < 0.05.

In order to further analyze the crAssphage dynamics, we examined phage richness and diversity for the crAssphage family and separated them into each subfamily. The analysis at the family level showed that phage diversity and richness decreased in O and OMS compared to NW (Figure 4a,b). However, the differences among the groups were not significant. Notably, when we analyzed the subfamily level, we found that the diversity (*p* = 0.018) and richness (*p* = 0.018) of Alpha showed a significant decrease in OMS when compared to NW (Figure 4c,d). We also performed a β-diversity analysis to understand if crAssphage clustering was associated with the O and OMS groups; however, no clustering was observed.

### 3.4. Host Bacteria of crAssphages Were Decreased in the O and OMS Groups

**Figure 5:**
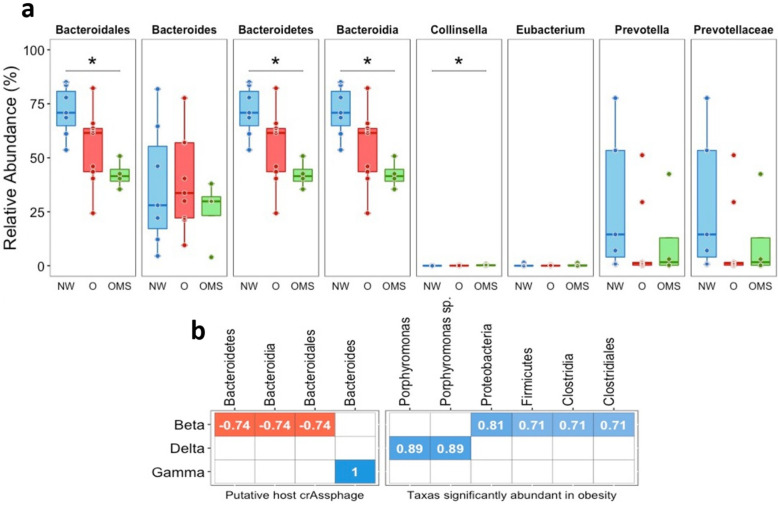
(**a**) Relative abundance of putative bacterial crAssphage hosts. Each point shows the abundance for each sample, with the boxes showing the group’s distribution. Error bars represent the median ± SD. The p-value resulting from comparisons among groups is shown above the box plots, *: *p* < 0.05. (**b**) Spearman correlation plot between the abundances of bacteria and crAssphage subfamilies.

Next, we studied the abundances of the putative crAssphage hosts using 16s rRNA amplicon sequencing data. To this end, we analyzed the abundance of the Bacteroidetes, Bacteroidia, Bacteroidales, Prevotellaceae, Prevotella, Collinsella, Bacteroides, Bacilli, and Eubacterium, which were previously suggested to be the bacterial hosts of crAssphages [11,16,19,22,27]. We observed that Bacteroidetes, Bacteroidia, and Bacteroidales showed a significant decrease in OMS compared to NW (*p* = 0.006), while Collinsella showed significant augmentation in OMS compared to NW (*p* = 0.04). (Figure 5a). The other groups of the analyzed bacteria presented no significant changes. Prevotella and Prevotellaceae were more abundant in NW compared to O and OMS, while Eubacterium was more abundant in OMS than in NW and O. Next, we assessed whether the abundance of the crAssphage subfamily correlated with the abundance of putative host bacteria. To this end, we selected the above mentioned putative host bacteria and calculated the Spearman correlation of their abundance against the abundance of all crAssphages genomes in all samples. However, we do not find any significant correlation. Interestingly, when we compared the crAssphage abundances grouping by each subfamily against the putative host bacteria, we found that the Beta and Gamma subfamilies correlated with putative bacteria hosts (Figure 5b).

### 3.5. Increased Bacilli Were Associated with Decreased crAssphage Abundance in OMS

**Figure 6:**
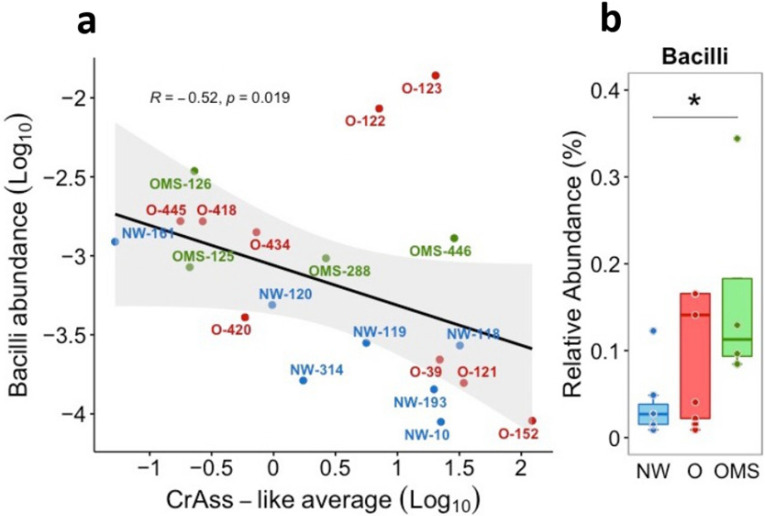
Relative abundance and Spearman correlation between the Bacilli and crAssphage genomes. (**a**) Spearman correlation plot between the Bacilli and crAssphage abundances. Blue circles = NW samples; red circles = O samples; green circles = OMS samples. (**b**) Relative abundance (%) of Bacilli. Each point shows the abundance for each sample, with the boxes showing the group’s distribution group (* *p* = 0.024).

We next studied the relationship between the crAssphage family and bacterial increases in O and OMS [26]. To do this, we calculated each sample’s Spearman correlation between the abundance of the 27 taxa previously associated with O and the crAssphage family. We only found one significant negative correlation between crAssphage abundance and the Bacilli (Figure 6a). Notably, the Bacilli also significantly increased in OMS compared to NW (Figure 6b). Next, we also analyzed the association between the crAssphage subfamilies and the 27 taxas and found several significant correlations between the Beta, Delta, and Gamma subfamilies (Figure 5b).

Finally, we also analyzed whether crAssphage abundance correlated with the anthropometric and clinical parameters typically altered in O and OMS, such as a high body mass index (BMI), low levels of high-density lipoprotein (HDL), high levels of triglycerides, a high glucose level, a wide waist circumference, and high weight [26] However, we did not find any significant correlation.

## 4. Discussion

To the best of our knowledge, this is the first report detailing the role of different crAssphage subfamilies in a cohort of children with O and OMS. Until now, there are two previous studies that are closely related. The first described the abundance of the crAssphage family in the adult fecal samples of patients with metabolic syndrome using qPCR [28]. This study observed a statistically and significantly high presence of crAssphage in metabolic syndrome patients compared to healthy controls (*p* < 0.05). Furthermore, the second study performed total DNA and VLPs sequencing in 196 adult patients with metabolic syndrome [29], observing that the relative abundance of crAssphage did not significantly differ between the metabolic syndrome patients and the controls. In contrast, our children’s cohort showed a decreased abundance of the Alpha crAssphage subfamily in the OMS group (particularly genera Alpha_1 and Alpha_4). Moreover, it is worth mentioning that, due to infrastructure and cost limitations, the collected data can be considered a small sample size: 10 normal weight (NW), 10 obese (O), and eight obese with metabolic syndrome (OMS) children. Nevertheless, our data allowed for adequate statistical analyses that showed significant differences in the results.

The quantitative analysis of the crAssphage genomes revealed that most of our samples contained varying amounts of crAssphage genomes, from 0.0011 to 3.654% of the total reads. Similar crAssphage abundances also were reported in healthy Malawian infants [7]. For the first time, our work also provides significant differential abundance of specific crAssphage taxa associated with O and OMS. Specifically, the Alpha subfamily and Alpha_1 and Alpha_4 genera were found to have decreased in OMS, contrary to a significant increase in Delta_8.

A loss of stability in the crAssphage Alpha subfamily and an increase in the Delta subfamily were associated with O, being significant in OMS. Thus, the decreased abundance of Alpha and the increased abundance of Delta bacteriophages are associated with OMS. This is a description of the double-faced role of the crAssphage family in O and OMS: between good and evil. However, the exact mechanism behind this trade-off needs further elucidation.

The crAssphage Alpha subfamily was more abundant in the NW than OMS individuals, suggesting that these bacteriophages could be used as a promising treatment for obesity and metabolic syndrome through their fecal transplantation. In addition, a couple of studies support the engraftment of crAssphages [19,30]. For example, Siranosain et al. [19] observed that crAssphages are rarely detected at birth but are increasingly prevalent in the microbiome after one month. Interestingly, the crAssphage genomes found in these infants were nearly identical to the ones found in their mothers in 50% of cases, suggesting vertical transmission [19]. Notably, this study reports that one-year-old infants had an average relative abundance of 0.011% of crAssphage.

In comparison, our cohort of 7–10-year-old children showed slightly lower abundances (0.005%), which could be due to natural variability among populations. Furthermore, Draper et al. [30] also observed that crAssphage can be transmitted after fecal transplantation and gradually dominate the virome without affecting the levels of their host populations [30]. Indeed, supplemental phage intake had no significant impact on overall health status and gut microbiota, and only specific bacteria were altered [31].

Recently, phages were administered as a dietary supplement in healthy and gastrointestinal distressed individuals without causing exacerbation to their symptoms [32]. Additionally, crAssphages use an unusual strategy to establish themselves in the gut and stably persist within the microbial communities for several weeks and months [2]. Interestingly, the use of Quyushengxin, a traditional herbal Chinese formula, showed decreased diversity for the crAssphage family in patients with ulcerative colitis, although no other mechanisms were explored [25]. This suggests that crAssphages could be modulated using pharmaceutical or natural treatments. However, care must be taken in the double-face role of the crAssphage family because the Delta subfamily was more abundant in OMS, so if they are also transplanted it could worsen the bacterial dysbiosis in OMS.

A recent analysis of 1135 individuals found no significant association between crAssphage abundance and health/diseases. The study examined the correlation between crAssphage abundance and 207 human variables, including 39 diseases [13]. Other studies showed no significant differences in crAssphage abundance in fecal samples between diarrhea and healthy adults [16]. Compared to normal controls, a significant increase in crAssphage abundance was reported in patients with ulcerative colitis [25]. A recent study showed decreased crAssphage abundance due to colorectal cancer compared to healthy controls, suggesting a promising treatment using fecal crAssphage transplantation for this disease [24]. In line with this, we found a decrease in the Alpha subfamily’s abundance, richness, and diversity in the obese and metabolic syndrome groups. We also observed a decreased prevalence of this subfamily in the OMS. A previous study of the human phageome also found decreased abundance for crAssphage in O and OMS compared to normal weight [8].

The significantly increased abundance of Bacilli and its families *Streptococcaceae* and *Lactobacillaceae* was reported in O across two independent and large American adult populations [33]. Notably, we found that *Bacilli* had also significantly increased in OMS when compared to NW. Furthermore, a negative correlation was found between the abundance of crAssphages and the order *Bacilli*, suggesting that a decrease in crAssphages could be associated with a significant increase in *Bacilli.*

Bacteroidetes was initially proposed as the host phylum for crAssphages [11]. Further investigations have also linked Bacteroidales, Collinsella, Bacteroides, Bacteroidia, and Prevotellaceae with putative host bacteria [11,16,19,22,27]. Specifically, two crAssphages have been isolated in pure culture: ΦcrAss001 and ΦcrAss002, infecting Bacteroides intestinalis and Bacteroides Xylanisolvens, respectively [22,27]. In this regard, we tested if our samples’ abundance of the proposed host bacteria of crAssphages correlated with changes in crAssphage abundance. From the putative host bacteria of the crAssphages, we only found a significantly negative correlation between the abundances of the Beta subfamily with Bacteroidetes, Bacteroidia, and Bacteroidiales and between a significantly positive correlation between the Gamma subfamily and Bacteroides (Figure 6). A correlation between the abundance of crAssphage and Bacteroides using 16S rRNA data was reported [34]. It is important to note that crAssphages also replicate in a way that does not disrupt the proliferation of the host bacterium, maintaining itself in the continuous host culture for weeks, suggesting their ability to maintain the stable colonization of the mammalian gut [2]. Indeed, the persistent propagation of virulent phages can occur in the human gut without host elimination [27]. Nonetheless, it is interesting that a significant decrease in the abundance of Bacteroidetes, Bacteroidia, and Bacteroidales was observed in O and OMS.

## Data Availability

The sequence data used in this study is available in the NCBI under the NCBI BioProject accession number: PRJNA646512.

## Supplementary Materials

The following supporting information can be downloaded at: https://www.mdpi.com/article/10.3390/genes14010139/s1, Table S1: Database of crAssphage genome genomes, Table S2. Genomes of bacteriophages used as Non-intestinal phage controls, Table S3: Samples selected for the study, Table S4: Mean percentage (%) of total reads mapped to the crAssphage genome database.
